# A short peptide protects from age‐onset proteotoxicity

**DOI:** 10.1111/acel.14013

**Published:** 2023-10-27

**Authors:** Hassan Elsana, Reut Bruck‐Haimson, Huadong Zhu, Atif Ahmed Siddiqui, Adam Zaretsky, Irit Cohen, Hana Boocholez, Noa Roitenberg, Lorna Moll, Inbar Plaschkes, David Naor, Ehud Cohen

**Affiliations:** ^1^ The Lautenberg Center of Immunology and Cancer Research The Institute for Medical Research Israel – Canada (IMRIC), The Hebrew University School of Medicine Jerusalem Israel; ^2^ Department of Biochemistry and Molecular Biology The Institute for Medical Research Israel – Canada (IMRIC), The Hebrew University School of Medicine Jerusalem Israel; ^3^ Info‐CORE Bioinformatics Unit of the I‐CORE, The Hebrew University Jerusalem Israel

**Keywords:** aging, *C*. *elegans*, neurodegeneration, proteostasis, trans chaperone signaling

## Abstract

Aberrant protein aggregation jeopardizes cellular functionality and underlies the development of a myriad of late‐onset maladies including Alzheimer's disease (AD) and Huntington's disease (HD). Accordingly, molecules that mitigate the toxicity of hazardous protein aggregates are of great interest as potential future therapeutics. Here we asked whether a small peptide, composed of five amino acids (5MER peptide) that was derived from the human pro‐inflammatory CD44 protein, could protect model nematodes from the toxicity of aggregative proteins that underlie the development of neurodegenerative disorders in humans. We found that the 5MER peptide mitigates the toxicity that stems from both; the AD‐causing Aβ peptide and a stretch of poly‐glutamine that is accountable for the development of several disorders including HD, while minimally affecting lifespan. This protection was dependent on the activity of aging‐regulating transcription factors and associated with enhanced Aβ and polyQ35‐YFP aggregation. A transcriptomic analysis unveiled that the peptide modifies signaling pathways, thereby modulating the expression of various genes, including these, which are known as protein homeostasis (proteostasis) regulators such as *txt‐13* and modifiers of proteasome activity. The knockdown of *txt‐13* protects worms from proteotoxicity to the same extent as the 5MER peptide, suggesting that the peptide activates the transcellular chaperone signaling to promote proteostasis. Together, our results propose that the 5MER peptide should be considered as a component of future therapeutic cocktails for the treatment of neurodegenerative maladies.

## Graphical abstract

Short peptides have been shown to mitigate protein aggregation. We report here that the peptide MTADV protects model nematodes from the aggregation of toxic proteins that cause neurodegeneration in humans. This protection is conferred by the modulation of gene expression and the enhancement of protective protein aggregation. Accordingly, this peptide should be considered as a component of future therapeutic cocktails for the treatment of neurodegenerative maladies.

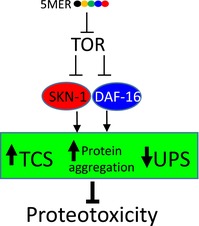

**Abbreviations**
AD: Alzheimer's disease
APP: Amyloid precursor protein
Aβ: Amyloid β
EV: Empty vector
GFP: Green fluorescent protein
HD: Huntington's disease
HSF‐1: Heat shock factor‐1
NGS: Next generation sequencing
PN: Proteostasis network
PolyQ: Poly‐glutamine
Proteostasis: Protein homeostasis
Proteotoxicity: Toxic protein aggregation
qPCR: Quantitative real‐time PCR
RA: Rheumatoid Arthritis
RNAi: RNA interference
RNA‐seq: RNA sequencing
SAA: Serum amyloid A
WB: Western blot
YFP: Yellow fluorescent protein
SCF: Stem cell factor
TCS: Transcellular chaperone signaling
TOR: Target of rapamycin
UPS: Ubiquitin proteasome system

## 1 INTRODUCTION

Maintaining the integrity of the proteome is crucial for cellular and organismal functionality and viability. To achieve and maintain protein homeostasis (proteostasis), organisms have developed a network of mechanisms that act in an orchestrated manner to assist nascent polypeptides in reaching their functional spatial folding, ensure accurate protein–protein interactions, and degrade terminally misfolded proteins by specialized mechanisms (Jayaraj et al., 2019). Early in life, these coordinated activities of the “proteostasis network” (PN) successfully ensure proteostasis; however, as the organism ages, proteins that bear an intrinsic propensity to misfold escape the cellular surveillance mechanisms and form insoluble aggregates that accrue within cells and tissues. The accumulation of protein aggregates jeopardizes proteostasis and can lead to the development of a myriad of diseases that are collectively known as “proteinopathies” (Paulson, 1999). Late‐onset neurodegenerative diseases are prevalent proteinopathies that affect elder individuals throughout the industrialized world. Alzheimer's disease (AD) (Long & Holtzman, 2019) and poly‐glutamine (polyQ) expansion disorders, such as Huntington's disease (HD) (Shao & Diamond, 2007), are incurable neurodegenerative maladies that stem from toxic protein aggregation (proteotoxicity) (Carvalhal Marques et al., 2015).

The aggregation of a family of peptides known as “Amyloid β (Aβ) peptides” underlies the development of AD. Aβ peptides are cleavage products of the type 1 transmembrane protein, amyloid precursor protein (APP), which undergoes digestion by two proteolytic entities, the β and γ secretases. The “Amyloid hypothesis” (Hardy & Selkoe, 2002) proposed that an elevated digestion of APP leads to an increased production of the family of Aβ peptides. These peptides aggregate and accumulate in plaques, which in turn, underlie the manifestation of AD. This hypothesis was the basis for most attempts to develop remedies for AD, alas; the amyloid hypothesis fails to explain how familial (Ben‐Gedalya et al., 2015) and sporadic (Szaruga et al., 2015) cases of AD onset. Accordingly, all efforts to develop remedies for AD, have failed (Panza et al., 2019). In fact, despite the recent approval of the antibody lecanemab as a new treatment for AD, its safety and efficacy are still questionable (Couzin‐Frankel, 2023).

Similarly, abnormally long poly‐glutamine expansions render different proteins to be aggregation‐prone and underlie the development of various neurodegenerative disorders. These include Huntington's disease and different types of ataxia, all of which are incurable (Shao & Diamond, 2007).

The lack of treatment for these devastating diseases calls for the development of new therapeutic compounds which could be combined to create drug cocktails that concurrently modulate different cellular and organismal mechanisms to efficiently promote proteostasis, postpone the development of neurodegenerative disorders, and slow their progression once emerged.

Here we tested whether a novel 5MER peptide, MTADV (Methionine Threonine, Alanine, Aspartic Acid, and Valine), is capable of mitigating neurodegenerative‐causing proteotoxicity. The 5MER peptide was originally derived from a sequence of a pro‐inflammatory CD44 variant isolated from the synovial fluid of a rheumatoid arthritis (RA) patient. This human peptide displays an efficient anti‐inflammatory effect and ameliorates pathology and clinical symptoms in mouse models of multiple sclerosis, RA, and inflammatory bowel disease. The 5MER peptide was also found to disrupt the assembly of oligomers and aggregates of the serum amyloid A protein (SAA), which are correlated with the SAA pro‐inflammatory activity, leading to chronic inflammation and amyloidosis (Hemed‐Shaked et al., 2021). These counter‐proteotoxic properties of the 5MER peptide have led us to ask whether it is capable of mitigating the toxicity of neurodegeneration‐causing amyloidegenic peptides. To address this, we employed transgenic *Caenorhabditis elegans* (*C*. *elegans*) nematodes that were engineered to express aggregative peptides known to cause neurodegenerative diseases in humans. We found that the 5MER peptide protects the worms from motility impairments that emanate from the expression of Aβ or polyQ stretches in muscles and neurons. This protection was entirely dependent on the expression of proteostasis‐promoting transcription factors (Cohen et al., 2006; van Oosten‐Hawle et al., 2013), and was associated with enhanced protective protein aggregation. Using RNA sequencing (RNA‐seq), we characterized the gene networks that are influenced by the 5MER peptide and discovered that the transcriptomic landscape is more significantly modulated upon exposure to the peptide in worms that express high Aβ levels compared to the changes that were observed in their counterparts that express very low levels of this aggregative peptide. Our data also suggest that the 5MER peptide modulates the activity of the target of rapamycin (TOR) kinase. Among the genes that exhibited increased expression levels upon treatment with the 5MER peptide, we identified several nuclear hormone receptors and a gene that codes for a protein that is involved in the mediating trans‐chaperone signaling.

Our findings indicate that the 5MER protects model nematodes from proteotoxicity by modulating the activity of the PN and suggest that this peptide could be a component of future therapeutic cocktails for the treatment of neurodegenerative disorders.

## 2 MATERIALS AND METHODS

### 2.1 Peptides

The 5MER peptide (MTADV) was produced by Sigma‐Aldrich Rehovot, Israel and Sigma‐Aldrich Woodlands, TX, USA (>95% purity). 5MER and the scrambled peptides (TMVAD) were also produced by PepTech Corp., Burlington, MA, USA (>98% purity). The peptides were acetylated at the N‐terminus and amidated at the C‐terminus by the manufacturers to improve stability.

### 2.2 *C*. *elegans* strains and growth conditions

N2 (wild‐type, Bristol), CL2006 (*unc‐54*p::human Aβ_3‐42_) and AM140 (*unc‐54*p::polyQ35‐YFP) worms were obtained from the Caenorhabditis Genetics Center (CGC), which is funded by the NIH Office of Research Infrastructure Programs (P40 OD010440). AM716 worms (*rgef‐1p*::polyQ67‐YFP) were a generous gift from Prof. Richard Morimoto (Northwestern University). CF512 (fer‐15(b26)II; fem‐1(hc17)IV), daf‐16(mu86), muIs109 [Pdaf‐16::gfp::daf‐16cDNA + Podr‐1::rfp] (CF1934), and glp‐1(e2144ts) strain (CF1930) were generously provided by Prof. Andrew Dillin (Berkeley University). N2, CL2006, CF1934, AM140, and AM716 strains were grown at 20°C on nematode growth media (NGM) plates and fed with *Escherichia Coli* HT115 bacteria. CF512 and CF1930 animals are heat‐sensitive and thus were routinely grown at 15°C. To avoid progeny, CF512 and CF1930 worms were hatched at 20°C, and L1 larvae were transferred to 25°C for 48 h and back to 20°C. Standard *C*. *elegans* techniques were used to maintain the strains.

### 2.3 Paralysis and thrashing assays

For the paralysis assay, synchronized CL2006 eggs were placed on NGM‐ampicillin plates seeded with the desired bacteria and supplemented with 100 mM ampicillin (4 mM final). The worms were allowed to develop until day 1 of adulthood. One hundred and twenty animals were then transferred onto small NGM plates seeded with the respective *E*. *coli* culture. Paralyzed worms were recorded daily by tapping the worms' noses with a platinum wire. A paralyzed animal was defined as an animal than can move its head but is unable to crawl away. The assays were terminated at day 12 of adulthood, when the wild‐type animals started exhibiting age‐related paralysis. The trashing rate was determined by transferring individual animals into a drop of M9 buffer at the indicated time points, according to the strain (AM140 or AM716). The worms were allowed 30 s for adaptation, and the number of body bends was counted during the next 30 s. Twenty animals per treatment per independent experiment were used, and at least three independent experiments were performed.

### 2.4 Lifespan assays

Synchronized eggs were placed on master NG‐ampicillin plates seeded with the indicated RNAi bacterial strain and supplemented with 100 mM IPTG (~1 mM final). CF512 worms were hatched at 20°C, transferred to 25°C for 48 h to avoid progeny, and back to 20°C. At day 1 of adulthood, 120 animals per treatment were transferred onto small NG‐ampicillin plates (12 animals per plate). Worms that failed to move their tips when tapped twice with a platinum wire were scored as dead.

### 2.5 Western blot of Aβ

Ten thousand worms were treated with 200 μM 5MER peptide and homogenized using a glass Dounce homogenizer (Kimble cat # 885301‐0002). The worm homogenates were spun for 3 min at 850 *× g* (3000 rpm in a desktop centrifuge) to sediment debris. The post‐debris supernatants (PDS) were collected, protein amounts were measured by a BCA kit, supplemented with loading buffer (10% glycerol, 125 mM Tris base, 1% SDS), boiled for 10 min and 20 μg of total protein were loaded into each well. Proteins were separated by sodium dodecyl sulfate polyacrylamide gel electrophoresis (SDS‐PAGE), transferred onto a nitrocellulose membrane (#66485; Pall Corporation), and probed with an Aβ antibody (#SIG‐39320; clone 6E10, Covance). HRP‐conjugated secondary antibodies, a chemiluminescence system, and a luminescent image analyzer (Chemidoc XRS+, Biorad) were used to detect protein signals.

### 2.6 Native agarose gel electrophoresis (NAGE)

AM140 worms were either soaked with 200 μM 5MER at days 1 and 2 of adulthood or left untreated. Both worm groups were homogenized at day 3 of adulthood (4°C). For each sample, 100 μg of total protein were loaded onto a 1% agarose gel and run at 4°C, 40 V for 15 h. YFP fluorescence intensities in the gels were visualized using a Typhoon FLA 9500 (GE Healthcare).

### 2.7 Filter trap assay

AM140 worms were grown on HT115 bacteria harboring the empty RNAi vector (EV). On days 1 and 2 of adulthood, the animals were soaked for 3 h with 200 μM 5MER peptide. At day 3, the worms were washed with M9 buffer, collected, and frozen in liquid N2. The worm pellets were thawed on ice and disrupted using a bullet blender in a lysis buffer (50 mM Hepes pH 7.4, 150 mM NaCl, 1 mM EDTA, 1% Triton X‐100) supplemented with an EDTA‐free protease inhibitor cocktail (Roche). Debris were sediment by low‐speed centrifugation (8000 × *g* spin for 5 min, 4°C). 100 μg of each protein extract was supplemented with SDS to the final concentration of 0.5% and loaded onto a cellulose acetate membrane assembled in a slot blot apparatus (Bio‐Rad cat#1703938). The membrane was washed with 0.2% SDS, and the retained Q35‐GFP was assessed by immunoblotting for GFP.

### 2.8 Computational analysis of polyQ35‐YFP foci

To determine the size distribution of foci, we used the ImageJ software. Automatic threshold was determined, which converted the image into a binary image. The particles were filtered by area to include ones between 15 and 1000 pixels^2^. These values ensure excluding of noise and large particles deriving from continuous labeling. The same procedure was used to analyze all images from all time points and treatments.

### 2.9 RNAi and cloning procedures

All RNAi experiments were carried out on NGM‐ampicillin plates seeded with *E*. *coli* harboring the appropriate RNAi clone grown in LB medium overnight at 37°C and supplemented with 100 mM isopropyl β‐d‐1‐thiogalactopyranoside (IPTG; final concentration of 4 mM) to induce the expression of the dsRNA. Empty vector (pAD12) and *daf‐16* (pAD43) RNAi were a generous gift of Prof. Andrew Dillin. *skn‐1*, *hsf‐1* and *pqm‐1* RNAi were obtained from the library generated in the laboratory of Marc Vidal. *txt‐13* and *nhr‐58* RNAi plasmids were created by amplifying the relevant sequences (Table S2) using PCR and cloning into the L4440 plasmid using the restriction enzymes Nhl‐1 and Xho‐1.

### 2.10 RNA isolation, next generation sequencing and quantitative real‐time PCR

Total RNA was isolated using a NucleoSpin® RNA kit (MACHEREY‐NAGEL; 740955). For each time point, 10,000 synchronized eggs were placed on NG‐ampicillin plates seeded with HT115 bacteria. The worms were washed daily with M9 to get rid of progeny, and adult worms were transferred onto new plates. At day 6 of adulthood, the worms were harvested in M9 and frozen in liquid N_2_. The worms were thawed and subjected to mechanical disruption using a glass 2 mL Dounce homogenizer (Kimble cat # 885301‐0002). Homogenates were transferred to microcentrifuge tubes and centrifuged at 14,000 × *g* for 5 min. The supernatants were transferred to NucleoSpin® Filter (NucleoSpin® RNA kit; Macherey‐Nagel), and total RNA was purified according to the manufacturer's instructions. The RNA was quantified using a NanoDrop 2000c spectrophotometer.

*For NGS*, we used RNA ScreenTape kit (catalog #5067‐5576; Agilent Technologies), D1000 ScreenTape kit (catalog #5067‐5582; Agilent Technologies), Qubit® RNA HS Assay kit (catalog # Q32852; Invitrogen), and Qubit® DNA HS Assay kit (catalog #32854; Invitrogen). mRNA libraries were prepared using KAPA Stranded mRNA kit with mRNA Capture Beads (KAPA Biosystems, KK8421). In brief, 1 μg was used for the library construction; library was eluted in 20 μL of elution buffer. All DNA sample libraries were pooled into 10 nM samples. Multiplex sample pools were loaded on NovaSeq 6000 (Illumina) using NovaSeq 6000 SP Reagent Kit v1.5, 100 cycles (cat# 20028401), with 122 cycles of single‐end sequencing (data is available at GSE230640).

*For qPCR analysis*, cDNA was prepared by random‐priming, and cDNAs were generated by reverse transcription of the total RNA samples with iScript^RT^ Advanced cDNA Synthesis Kit for RT‐PCR (#170‐8891; Bio‐Rad, Hercules) per the manufacturer's protocol. Analyses by qPCR were performed with EvaGreen SuperMix (catalog #172‐5204; Bio‐Rad) and normalized to levels of *cdc‐42* cDNA. Primer sequences are listed in Table S2. The qPCR reactions for each gene were performed in triplicates.

### 2.11 Computational analyses of next generation sequencing data

Raw reads were processed for quality trimming and adaptor removal using fastx_toolkit v0.0.14 and cutadapt v2.10 (Marcel, 2011). The processed reads were aligned to the *C*. *elegans* transcriptome and genome version WBcel235 with annotations from Ensembl release 106 using TopHat v2.1.1 (Kim et al., 2013). Counts per gene quantification were done with htseq‐count v2.01 (Anders et al., 2015). Normalization and differential expression analysis were done with the DESeq2 package v1.36.0 (Love et al., 2014). Normalized counts were further batch corrected for the effect of the three repeats of the biological procedure. Pair‐wise comparisons were tested with default parameters (Wald test) without applying the independent filtering algorithm. Significance threshold was taken as *p*_adj_ < 0.1.

Wormcat (www.wormcat.com) was used for gene enrichment analysis.

### 2.12 In‐vitro proteasome activity assay

Worms were homogenized, and 10 μg of total protein per sample was loaded into a 96‐well plate and 25 μM of a Suc‐LLVY‐AMC (chymotrypsin‐like activity) fluorogenic substrate was added. The reactions were excited at 380 nm, and emission was measured at 480 nm every 5 min for 90 min at 25°C. Results at time 0 of each experiment were defined as base line.

### 2.13 Statistical analysis and software

The results are presented as the mean ± standard error of mean (SEM) of at least three independent, for qPCR experiments, paralysis, and thrashing assays, the statistical significance of differences was assessed using the Student's *t*‐test. All the statistical analyses and plotting of the data were performed using GraphPad Prism 9 (GraphPad Software, Inc.).

## 3 RESULTS

### 3.1 The 5MER peptide protects from proteotoxicity

**FIGURE 1:**
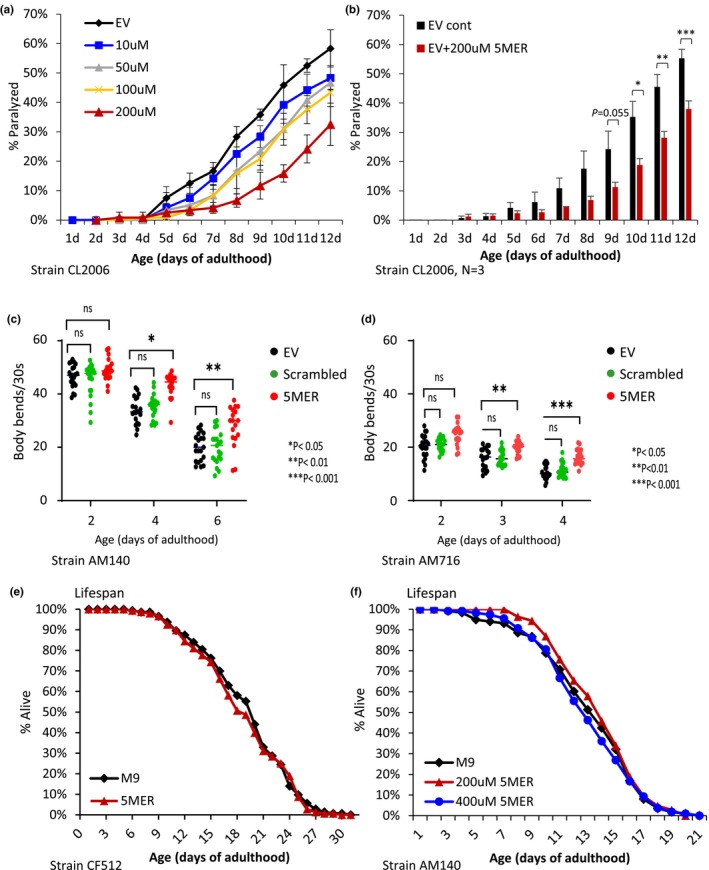
The 5MER peptide protects from proteotoxicity. (a) CL2006 worms were either left untreated (EV) or exposed to the indicated concentrations of the 5MER peptide (MTADV). A paralysis assay indicated that treatment with the peptide protects the worms from Aβ‐mediated paralysis in a dose‐response fashion. Treatment with 100 μM and with 200 μM showed significant protection (*p* = 0.019 and 0.0012, respectively). (b) Three independent experiments confirm that treatment with 200 μM 5MER peptide significantly protects the worms from paralysis (*N* = 3, *p < 0.05, **p < 0.01, ***p < 0.001). (c, d) The 5MER peptide mitigates the toxicity that emanates from poly‐glutamine (polyQ35‐YFP, strain AM140) stretches expressed in muscles (c) and protects from polyQ67‐YFP expressed in neurons (strain AM716) (d). (e) A daily exposure of CF512 worms to 200 μM 5MER peptide has no effect on lifespan. (f) AM140 worms were left untreated (M9) or treated with 200 or 400 μM 5MER peptide from day 1 of adulthood, and their lifespans were recorded. Treating the worms with 200 μM 5MER peptide slightly extended their lifespan by approximately 5.5% (*p* < 0.05). No lifespan extension resulted from a daily treatment with 400 μM 5MER peptide.

To test whether the 5MER peptide protects model worms from proteotoxicity, we employed animals that express the human Aβ_3–42_ peptide in their body wall muscles (strain CL2006). This expression, which is driven by the *unc‐54* promoter, impairs muscle function and results in progressive paralysis within the worm population (Link, 1995), a phenotype that serves as a measurement of proteotoxicity [paralysis assay (Cohen et al., 2006)]. Identical, two‐day‐old, synchronized populations of CL2006 worms were soaked for 3 h with 10, 50, 100, or 200 μM 5MER peptide. Thereafter, the same concentrations of the peptide were added daily to the plates during the entire experiment. An additional group of worms was soaked in the M9 buffer (EV) and served as a control group. Rates of paralysis within all worm populations were recorded daily. Treatment with 200 μM 5MER peptide provided the most efficient protection from proteotoxicity, as at day 12 of adulthood, 58% of the untreated animals were paralyzed, whereas only 33% of the animals that were exposed to 200 μM 5MER peptide showed this phenotype. Lower concentrations of the 5MER peptide have led to less prominent protection (Figure 1a). Three independent experiments confirmed the reproducibility and significance of the protection provided by 200 μM 5MER peptide (Figure 1b). Of note, treatment with higher concentrations of the 5MER peptide did not further reduce the rate of paralysis. In fact, treatment with 400 and 800 μM resulted in similar protection as 200 μM, and exposing the worms to 1000 μM abolished the protection (Figure S1A), suggesting that at high concentrations, the 5MER peptide may be toxic. In addition, treating the worms with 200 μM of a scrambled peptide, composed by the same amino acids but in a different order, did not protect the worms from Aβ (Figure S1B). Since 200 μM 5MER is the lowest concentration that significantly protects the worms from Aβ‐mediated toxicity, we continued our investigation using this concentration of the peptide.

We next asked whether the counter‐proteotoxic effect conferred by the 5MER peptide is Aβ‐specific or whether this peptide is capable of mitigating the proteotoxicity of other neurodegeneration‐causing aggregative peptides. To address this, we used worms that express poly glutamine (polyQ) stretches of 35 repeats fused to the yellow fluorescent protein (polyQ35‐YFP) in their muscles. Similarly to the Aβ worms, the expression of polyQ35‐YFP is regulated in these animals by the *unc‐54* promoter and leads to impaired muscle functionality (strain AM140; Morley et al., 2002). To measure the proteotoxicity that stems from polyQ35‐YFP, we utilized the thrashing assay (Volovik, Marques, & Cohen, 2014). Our results indicated that treating the worms with 200 μM 5MER peptide, but not with a scrambled peptide, slows the age‐associated decline in the number of body bends, indicative of protection from proteotoxicity (Figure 1c). Similar results were obtained when we employed worms that express polyQ67‐YFP in their neurons (strain AM716), showing that the 5MER peptide is also capable of protecting worms from aggregative polyQ‐YFP stretches that are expressed in neurons (Figure 1d).

While some compounds that promote health also extend lifespan (Alavez et al., 2011), these two features are not necessarily coupled (El‐Ami et al., 2014). Thus, we asked whether the 5MER peptide influences the lifespan of nematodes. To test this, we employed a synchronized population of heat‐sensitive feminized worms (strain CF512). The animals were exposed for 3 h to 200 μM 5MER peptide at day 1 of adulthood, placed on plates, and the same concentration of the peptide was supplemented to the plates daily. This exposure protocol, which protected CL2006 worms from proteotoxicity (Figure 1a,b), did not affect the lifespans of CF512 animals (Figure 1e and Table S4). Similarly, higher concentrations of the 5MER peptide, up to 1000 μM, did not significantly modify the lifespans of CF512 animals (Figure S1C), and 200 μM had no effect on the lifespans of wild‐type worms (Strain N2, Figure S1D). We also tested the possibility that the peptide affects the lifespans of worms that are challenged by proteotoxicity by recording the lifespans of AM140 worms that were treated with either 0, 200, or 400 μM 5MER peptide. We found that AM140 animals exhibit an approximately 30% shorter mean lifespan compared to CF512 worms (mean lifespans of 19.02 ± 0.434 and 13.2 ± 0.333 days, respectively; Figure 1f and Table S4). AM140 animals that were treated with 200 μM 5MER peptide showed a slight lifespan extension of 5.5% (mean lifespans of 13.96 ± 0.278 and 13.2 ± 0.333 days, respectively, *p* < 0.05). Nevertheless, no lifespan extension was observed when AM140 animals were treated throughout the experiment with 400 μM 5MER peptide. Together, these results indicate that while the 5MER peptide mitigates polyQ35‐YFP proteotoxicity, it has a marginal effect on lifespan and support the notion that lifespan and the maintenance of proteostasis are separable.

### 3.2 Enhanced aggregation in 5MER peptide‐treated worms

**FIGURE 2:**
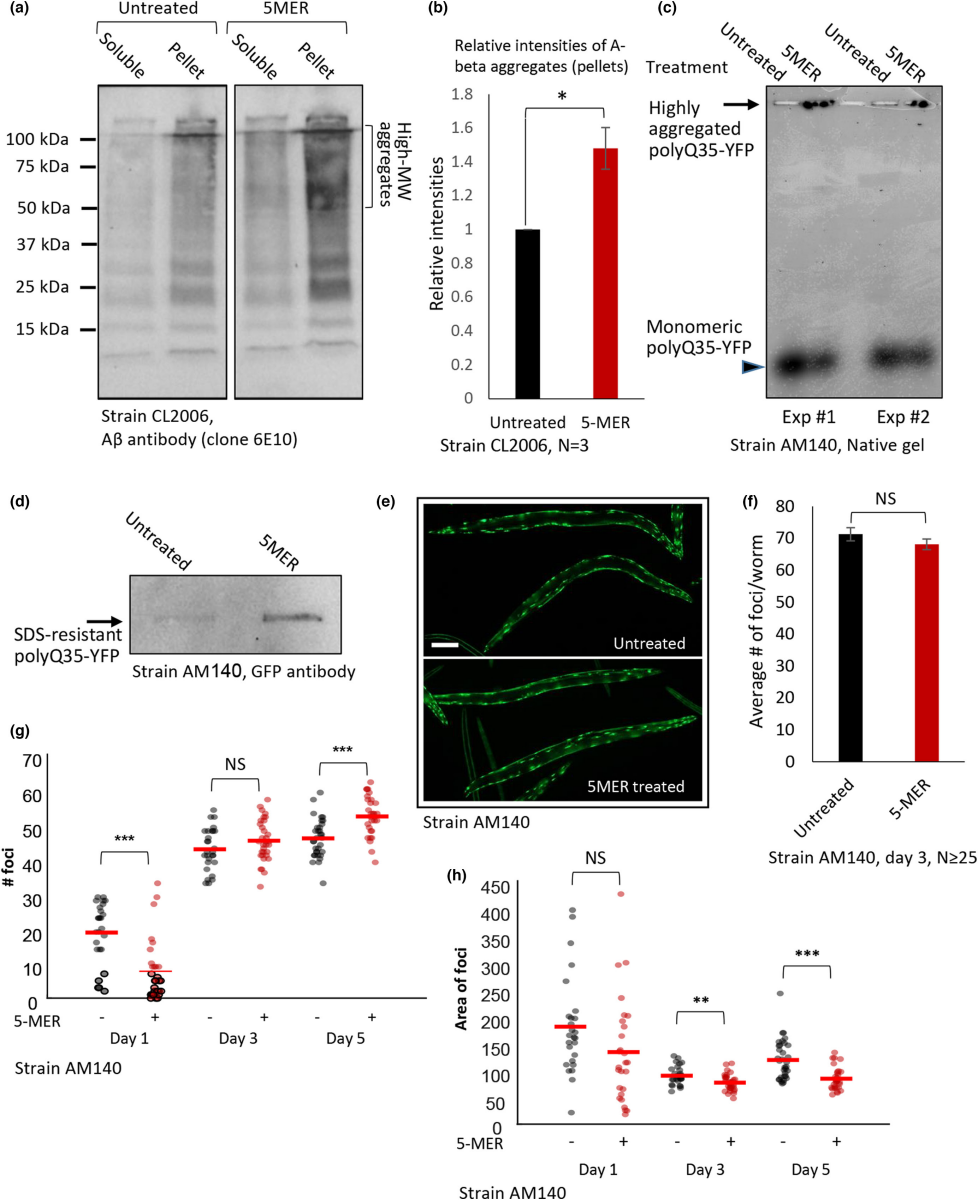
The 5MER peptide enhances protein aggregation. (a) Western blot analysis of CL2006 worms that were either left untreated of exposed to the 5MER peptide indicates that the peptide enhances the aggregation of Aβ. (b) Intensity quantification of three independent experiments as in (a). (c, d) Treatment with 200 μM 5MER peptide enhances the aggregation of polyQ35‐YFP as ascertained by native agarose gels (c) and by the filter trap assay (d). (e, f) The 5MER peptide does not affect the number of polyQ35‐YFP‐containing foci in 3 days old AM140 worms (e, scale bar 100 μm) as the average numbers are similar in treated and untreated animals (f). (g) A comparison of the numbers of polyQ35‐YFP‐containg foci in day 1, 3, or 5 old AM140 worms, unveiled that early in life (day 1) the 5MER peptide reduces the amount of foci, in day 3 it does not change it and in day 5 of adulthood a treatment with the peptide enhances the number of foci. (h) Computational analysis of foci size distribution shows that the 5MER peptide reduces the average area of polyQ35‐YFP foci in days 3 and 5 of adulthood.

Small oligomers, rather than large fibrils, have been reported to be the most toxic conformers of Aβ (Shankar et al., 2008) and of other neurodegeneration‐causing aggregative proteins (Silveira et al., 2005). Cells have developed two opposing ways to detoxify these highly toxic Aβ oligomers. One protective mechanism promotes the disaggregation and degradation of toxic oligomers, while the other assembles them to create large fibrils of lower toxicity (Cohen et al., 2006). Thus, we asked whether the 5MER peptide protects the worms by decreasing or enhancing the aggregation of Aβ. CL2006 worms were treated for 3 h with 200 μM 5MER peptide or soaked with M9 buffer (untreated) at days 1 and 2 of adulthood. At day 3 of adulthood, the animals were homogenized and spun to separate debris from soluble fractions. Equal protein amounts were separated on a gel, and Aβ was blotted. Our results indicated that the 5MER peptide enhances Aβ, aggregation as a higher rate of aggregates was observed in the pellets of treated worms compared to those of control animals (Figure 2a). This enhanced aggregation was reproducible and significant (Figure 2b).

We next tested whether the 5MER peptide also aggravates the aggregation of polyQ35‐YFP. AM140 worms were treated with the 5MER peptide as described above, homogenized at day 3 of adulthood, and polyQ35‐YFP aggregates were separated on a native agarose gel (Holmberg & Nollen, 2013). Two independent repeats indicated that the 5MER peptide increases the rate of polyQ35‐YFP aggregation, as highly aggregated conformers failed to enter the gel (Figure 2c, arrow). As expected, less soluble material was seen in these lanes compared to the levels observed in homogenates of untreated animals (Figure 2c, arrowhead). Similar results were obtained when we treated AM140 worms with the same regimen and compared the amounts of SDS‐resistant aggregated polyQ35‐YFP in control and 5MER‐treated animals using the “filter trap” assay” (Koyuncu et al., 2018; Figure 2d). These results confirmed that the peptide enhances the aggregation of polyQ35‐YFP in 3‐day‐old AM140 worms. Surprisingly, the enhanced aggregation rate of polyQ35‐YFP stretches conferred by the 5MER peptide was not associated with an elevated quantity of polyQ35‐YFP‐containing foci, as untreated day 3 old AM140 animals and their counterparts that were exposed to the 5MER peptide harbored similar numbers of foci (Figure 2e,f). To more comprehensively assess the effect of the 5MER peptide on foci formation in the context of age, we counted the number of these structures in AM140 worms of three ages, days 1, 3 and 5 of adulthood. We found that at day 1 of adulthood, the 5MER peptide reduces the amount of foci; at day 3, it does not affect it; and at day 5, it enhances foci formation (Figure 2g). This observation suggests that on days 3 and 5 of adulthood, the polyQ35‐YFP molecules deposited in these foci are in a higher aggregation state. To test this notion, we used a computational tool to compare the size distributions of polyQ35‐YFP‐containing foci in untreated and 5MER peptide‐treated worms and discovered that at days 3 and 5 of adulthood, treatment with the peptide results in foci of smaller size, proposing that polyQ35‐YFP in these foci is in a higher aggregation state (Figure 2h). Together, these results indicate that the 5MER peptide enhances the aggregation of Aβ and polyQ35‐YFP molecules and confirm that protection from proteotoxicity can be achieved by enhanced aggregation.

### 3.3 Proteostasis promoting transcription factors are needed for 5MER peptide‐mediated protection from Aβ

Aging‐regulating pathways, such as the insulin/IGF signaling (IIS) cascade, control lifespan (Kenyon et al., 1993), stress resistance (Honda & Honda, 1999), and proteostasis (Cohen et al., 2006; Morley et al., 2002). Upon activation, DAF‐2, the lone IIS receptor, initiates a signaling cascade that leads to the retention of a set of transcription factors in the cytosol. These include the forkhead factor DAF‐16/FOXO (DAF‐16), heat shock factor‐1 (HSF‐1), and the SKiNhead/Nrf (SKN‐1) factor (Carvalhal Marques et al., 2015). Accordingly, knocking down the activity of the IIS by *daf‐2* RNAi enables the entry of these transcription factors into the nucleus, where they regulate their target gene networks, thereby creating long‐lived, youthful worms (Kenyon, 2010). The ParaQuat Methylviologen Responsive (PQM‐1) factor is an additional IIS‐regulated transcription factor that changes its intracellular localization in opposition to DAF‐16. All these transcription factors have been reported to be pivotal proteostasis regulators (Blackwell et al., 2015; Cohen et al., 2006; O'Brien et al., 2018).

**FIGURE 3:**
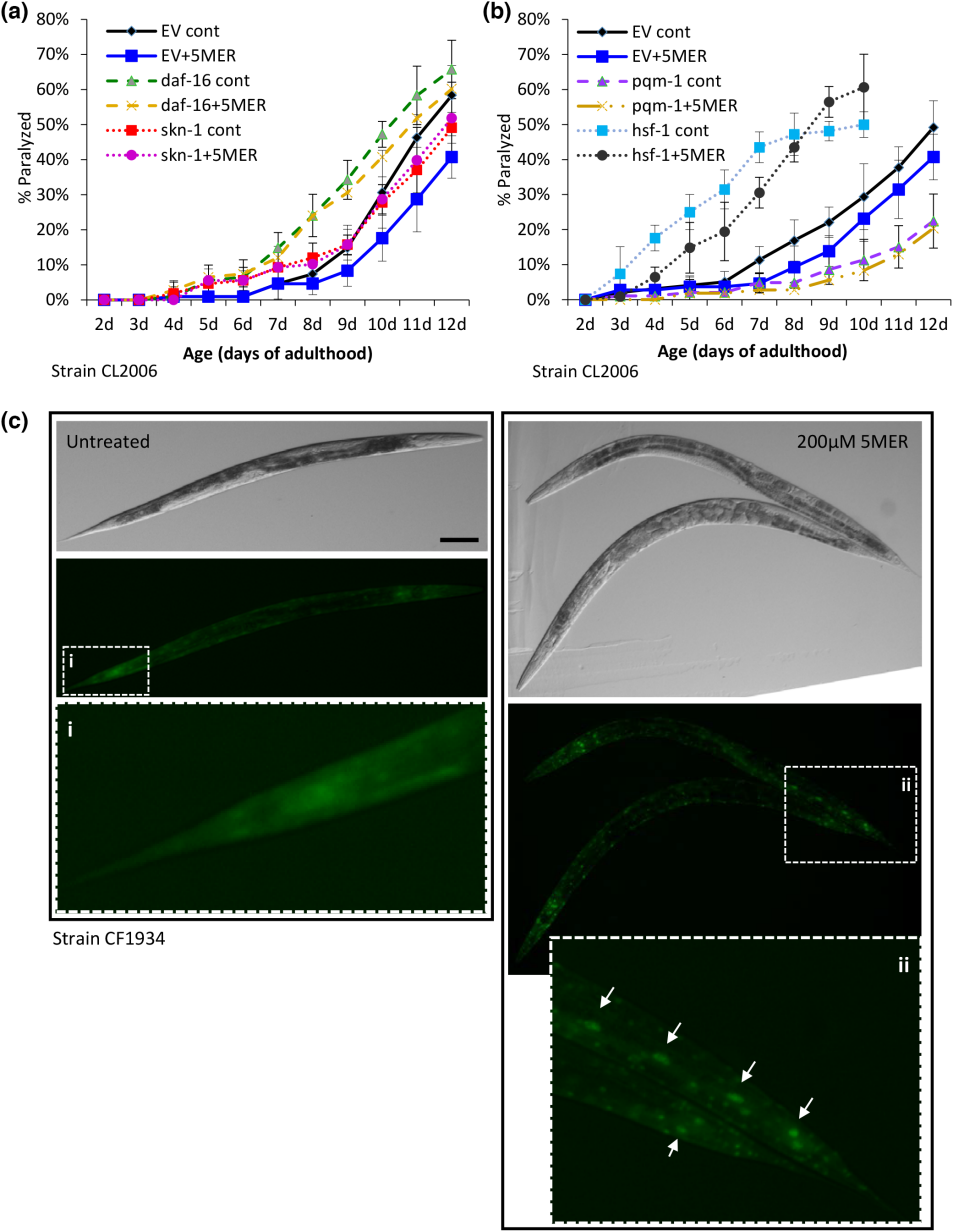
DAF‐16 and SKN‐1 are needed for the 5MER peptide‐mediated protection from Aβ toxicity. (a, b) Paralysis assays indicate that the 5MER peptide is incapable of mitigating Aβ toxicity when the expression of either *daf‐16* or *skn‐1* is knocked down by RNAi (a, *N* = 120 worms per treatment, *p* > 0.05 in all days). Treatment with the 5MER peptide provided partial protection despite the knockdown of *hsf‐1*. *pqm‐1* RNAi mitigates the toxicity that stems from Aβ but no additive effect was provided by the 5MER peptide (b, *N* = 120 worms per treatment, *p* > 0.05 at days 4, 6, and 7 for *hsf‐1* RNAi, but not for *pqm‐1* RNAi). (c) Exposure to the 5MER peptide drives GFP‐tagged DAF‐16 into the nuclei of CF1934 worms (inset arrows, scale bar = 100 μm).

To examine whether the counterAβ proteotoxicity properties of the 5MER peptide are dependent upon any of these proteostasis‐promoting factors, CL2006 worms were grown on bacteria that express RNAi toward either one of the aforementioned transcription factors or on EV bacteria. The worms were either treated with 200 μM 5MER peptide or soaked in the M9 buffer (untreated), and rates of paralysis in all worm groups were recorded daily. If a certain transcription factor is crucial for the 5MER peptide‐conferred protection from Aβ toxicity, we expected to observe no significant difference in the rates of paralysis of worms that were treated with RNAi toward this factor, regardless if they were exposed to the 5MER peptide or not. Of note, worms that were fed with RNAi bacteria toward *daf‐16* or *skn‐1* showed nearly identical levels of paralysis, unrelated whether they were treated with the 5MER peptide or not (Figure 3a). In contrast, the knockdown of *hsf‐1* enhanced paralysis compared to the control group; however, the 5MER peptide provided partial protection from this proteotoxic phenotype (Figure 3b). Intriguingly, the knockdown of *pqm‐1* mitigated Aβ‐mediated proteotoxicity to similar levels regardless of whether the worms were treated with the 5MER peptide or not (Figure 3b). This surprising observation, which suggests that this factor plays deleterious roles when Aβ is expressed, requires further validation and investigation.

The requirement of DAF‐16 for the 5MER peptide‐mediated protection from Aβ implies that this transcription factor enters the nucleus and regulates its target gene network upon treatment with the peptide. To directly test this notion, we utilized worms that express GFP‐tagged DAF‐16 under the regulation of the *daf‐16* promoter (strain CF1934). Day 1 old CF1934 worms were either soaked for 3 h with 200 μM 5MER peptide or left untreated, transferred onto plates for additional 6 h, and visualized thereafter by a fluorescent microscope. Our results clearly show that treatment with the 5MER peptide drives DAF‐16 into the nucleus (Figure 3c, arrows).

These results indicate that DAF‐16 and SKN‐1 are needed for the 5MER peptide‐mediated mitigation of Aβ toxicity and strongly suggest that the peptide modulates gene expression to enhance proteostasis.

### 3.4 Gene expression modulations by the 5MER peptide

**FIGURE 4:**
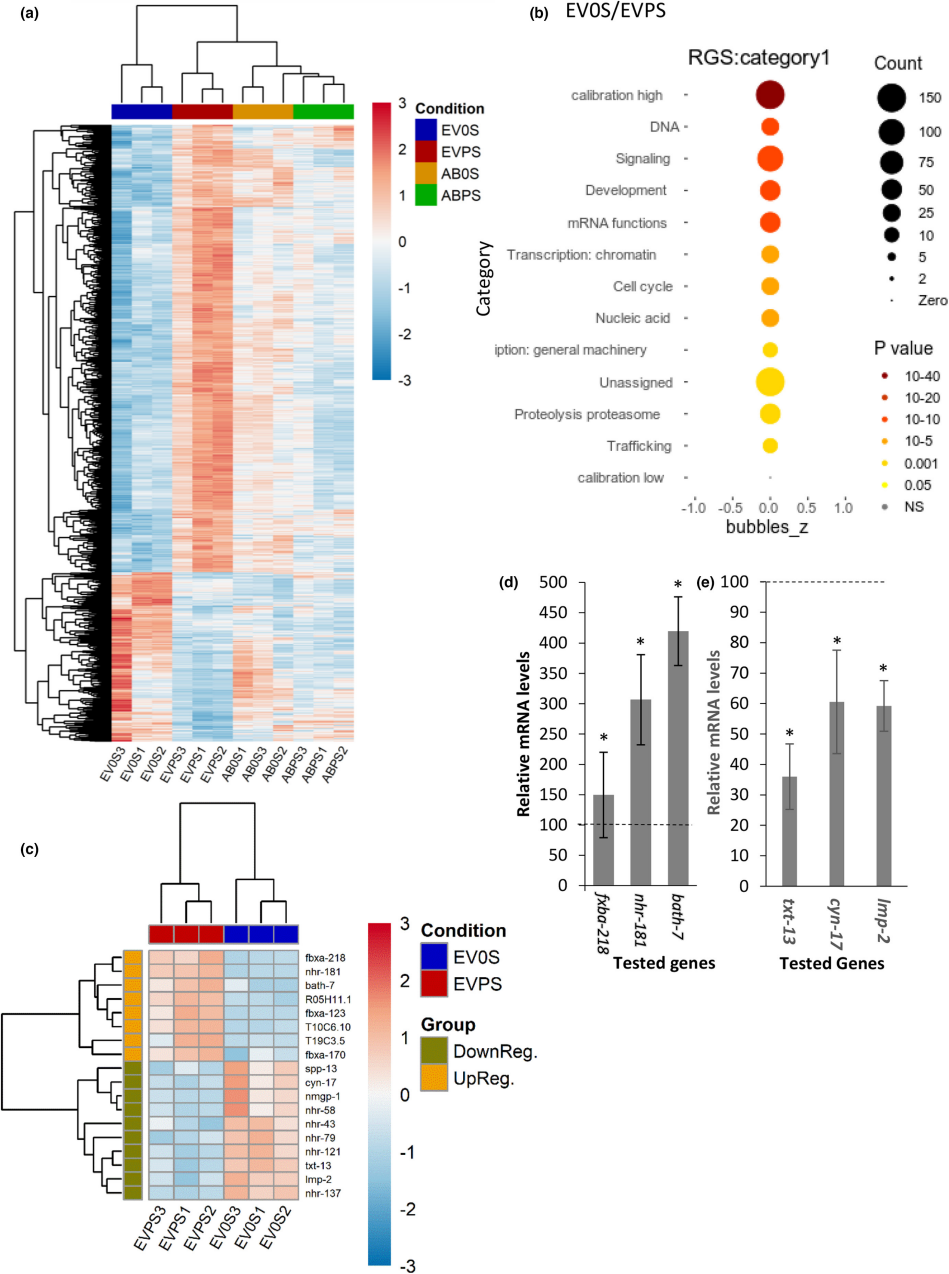
Analysis of gene expression modulation by the 5MER peptide in CL2006 worms. (a) A heat map displaying the overall expression pattern of differentially expressed genes (FDR corrected *p*‐value < 0.1) in day 6 old CL2006 worms that express high (EV0S and EVPS)) or low Aβ (AB0S and ABPS) levels and were either treated with 200 μM 5MER peptide or not. The displayed expression patterns indicates that the peptide more prominently affects gene expression in worms that express high Aβ levels (the levels of 1395 genes were modulated) compared to animals that were treated with Aβ RNAi (merely 301 genes exhibited significantly affected expression). (b) Enrichment analysis of the 1395 genes, using the Wormcat source, shows specific and significant modulated expression upon treatment with the 5MER peptide in worms that express high Aβ levels. Signaling, transcription and protein degradation are most prominently affected by the 5MER peptide in this analysis. (c) Nuclear hormone receptors, F‐box proteins and a modulator of trans‐chaperone signaling (*txt‐13*) are among the genes that exhibited significantly modulated expression levels upon treatment with the 5MER peptide. (d, e) qPCR using primers against a preselected gene groups that exhibited increased (d) or decreased (e) expression levels, validated the NGS results.

To test whether the 5MER peptide modifies gene expression and characterizes such modulations, we conducted an RNA‐Seq experiment to compare the transcriptomic landscapes of CL2006 worms that express high or low Aβ levels and were exposed to the 5MER peptide or left untreated. Four synchronized groups of CL2006 worms were included in the experiment: (i) animals that were grown on EV bacteria and were either exposed to the buffer (EV0S, control) or (ii) treated with the 5MER peptide (EVPS). These worms express Aβ uninterruptedly and thus, were challenged by high levels of proteotoxicity. (iii) Aβ RNAi‐treated CL2006 worms that were either exposed to the buffer (AB0S) or (iv) treated with the 5MER peptide (ABPS). In the two latter groups (iii and iv), the expression of Aβ is suppressed, and the animals are not challenged by a severe proteotoxic stress (Aβ RNAi reduces the levels of Aβ as shown by WB analysis in Figure S1E). This experimental setup enabled us to compare the effects of the 5MER peptide on gene expression in animals that are severely challenged by Aβ proteotoxicity to their counterparts that face minor proteotoxic stress. All worm populations were harvested at day 6 of adulthood, a post‐reproductive age when proteotoxicity is apparent, and RNA was extracted from three independent repeats. Gene expression in all groups was analyzed by Next Generation Sequencing (NGS, GEO accession number GSE230640). A comparison of gene expression modulations in worms that express high Aβ levels (EV0S and EVPS) and in those that express low Aβ levels (AB0S and ABPS) indicated that the 5MER peptide elicits much more comprehensive changes in gene expression when the worms are challenged by proteotoxicity. In fact, treatment with the 5MER peptide has led to significant (*p*_adj_ < 0.1) changes in the expression levels of 1395 genes in worms that express high Aβ levels (Figures 4a and S2A), but only 301 genes exhibited significantly modulated expression in animals that were treated with Aβ RNAi (Figure 4a). To characterize the changes elicited by the 5MER peptide, we used the Wormcat tool (http://www.wormcat.com/) and found that genes that are involved in signaling, transcription, and proteasome‐mediated protein degradation are prominent groups that show modulated expression upon treatment with the 5MER peptide in worms that are challenged by Aβ proteotoxicity (EV0S vs. EVPS, Figure 4b). Such modulations were not apparent in their counterparts that were grown on Aβ RNAi bacteria (AB0S and ABPS, Figure S2B), indicating that these changes occur due to proteotoxicity. A detailed analysis of the modulated genes that are involved in signaling pointed at Notch, WNT, and most prominently at TOR signaling pathways (Figure S2C,D) and suggested that their functions are affected by the 5MER peptide. To further test this notion, we conducted a paralysis assay using CL2006 to directly examine whether TOR and/or WNT signaling are involved in the protection from proteotoxicity that is conferred by the 5MER peptide. The worms were treated with RNAi toward either *lin‐44*, an orthologue of WNT, or *let‐363*, which codes for TOR. Our results (Figure S2E) show that the knockdown of *let‐363* protects the worms from Aβ‐mediated toxicity; however, a combined treatment with *let‐*363 RNAi and the 5MER peptide did not further protect the worms from proteotoxicity. This observation suggests that, as predicted by our RNA‐seq experiment, TOR signaling is reduced by the peptide. In contrast, *lin‐44* RNAi did not reduce proteotoxicity. However, the 5MER peptide mitigates proteotoxicity in *lin‐44* RNAi‐treated worms, showing that this gene is not needed for the counter‐proteotoxic effect of the peptide.

We further analyzed our RNA‐seq data by clustering the genes by molecular functions (Figure S3A, *p*_adj_ < 0.05) and found that kinase activity is the most prominent function of genes that show modulated expression upon treatment with the 5MER peptide in worms that expressed high Aβ levels. This finding further supports the theme that the peptide modifies signaling.

Focusing on genes that showed modulated expression upon exposure to the 5MER peptide in worms that express high Aβ levels, we also identified several genes that are predicted regulators of aging, proteostasis, or to be controlled by aging‐governing pathways (Figure 4c). Among them are *fbxa‐218*, *fxba‐123*, and *fbxa‐170*, which encode F‐box proteins. Members of this family of proteins have been reported to be components of the stem cell factor (SCF) ubiquitin‐ligase complexes (Zheng et al., 2002) that we found to be proteostasis regulators (Levine et al., 2019). The gene R05H11.1 is also predicted to code for an F‐box protein and was found to be regulated by DAF‐2. Nuclear hormone receptors consist of an additional prominent group of genes whose expression levels were affected by the 5MER peptide. While *nhr‐181* exhibited increased expression in 5MER‐treated worms, *nhr‐43*, *nhr‐58*, *nhr‐121*, and *nhl‐137* were downregulated (Figure 4c). The affinity of NHR‐121 to the estrogen receptor (Mimoto et al., 2007) suggests that the 5MER peptide reduces the expression of this gene to modulate signaling that originates from the reproductive system and controls proteostasis (Shemesh et al., 2013). Nevertheless, this speculation should be further scrutinized. The expression of *cyn‐17*, a gene that encodes a proline *cis/trans* isomerase of the cyclophilin family and is controlled by DAF‐2, DAF‐16, and SKN‐1, was also reduced by the 5MER peptide. Cyclophilins play key roles in protein folding, and mutations in the prion protein or in presenilin1 that prevent cyclophilins from assisting the folding of these proteins underlie the development of neurodegenerative disorders (Ben‐Gedalya et al., 2015). Using quantitative real‐time PCR (qPCR), we confirmed that three genes that were shown by the RNA‐seq to be upregulated and three that were expected to be downregulated exhibit the expected modulations in expression levels (Figure 4d,e). We further tested whether the expression levels of subsets of DAF‐16 (Murphy et al., 2003) and SKN‐1 (Oliveira et al., 2009) target genes were modified by the 5MER peptide and found that various targets of these transcription factors show changes in expression levels upon exposure to the peptide (Figure S3B,C, respectively). Similarly, the peptide changed the expression levels of a subgroup of chaperones; however surprisingly, the expression levels of certain proteostasis‐promoting chaperones, such as *hsp‐16*.*2*, were reduced (Figure S3D). To further scrutinize the effect of the 5MER peptide on *hsp‐16*.*2* expression, we utilized worms that express GFP under the *hsp‐16*.*2* promoter (strain CL2070). The worms were either treated with the 5MER peptide or left untreated, and the levels of GFP expression were compared by WB analysis. Our results show that, as predicted by the RNA‐seq experiment (Figure S3D), the peptide reduces GFP expression in these animals (Figure S3E).

**FIGURE 5:**
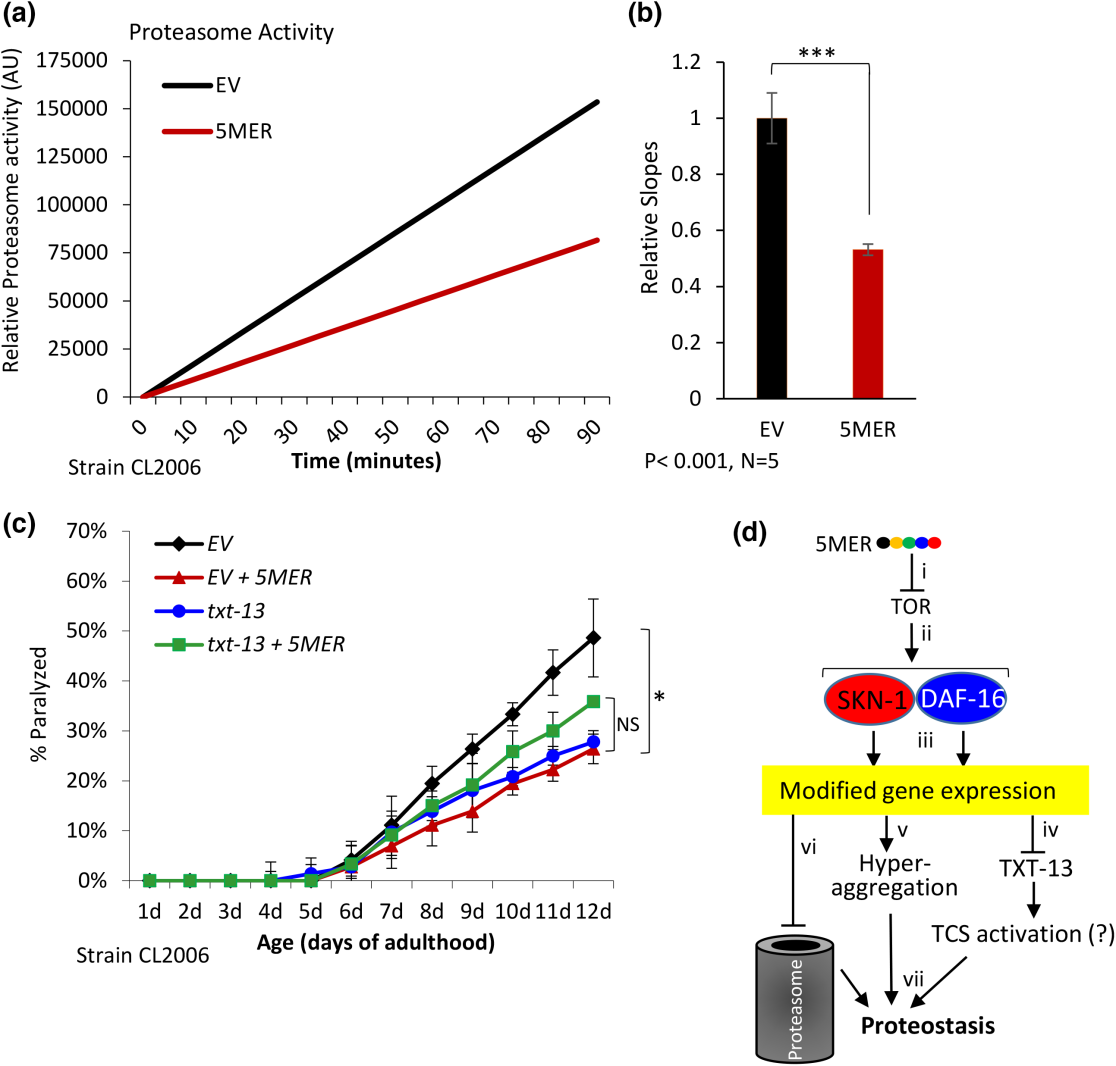
(a, b) The 5MER peptide reduces chymotrypsin‐like proteasome activity as measured by an in‐vitro assay (a). Five independent repeats confirm the significance of this observation (b). (c) The knockdown of *txt‐13* by RNAi mitigates the toxicity of Aβ, but a concurrent treatment with the peptide and *txt‐13* RNAi shows no additive protective effect (120 worms/treatment). (d) A proposed mechanism by which the 5MER peptide mitigates proteotoxicity. (i) The 5MER peptide inhibits the TOR kinase, (ii) thereby activates DAF‐16 and SKN‐1 (and perhaps other factors) to modulate gene expression (iii). This reduces the expression of *txt‐13* (iv) and activates TCS signaling, enhances protective protein aggregation (v) and reduces chymotrypsin‐like proteasome activity (vi). These modulations culminate to enhance proteostasis (vii).

Our RNA‐seq results predict that the 5MER peptide is involved in the regulation of proteasome activity and “transcellular chaperone signaling” (TCS) (Miles et al., 2023). To scrutinize these predictions, we first used CL2006 worms that were either treated with the 5MER peptide or not, homogenized them at day 6 of adulthood, and subjected the homogenates to an in‐vitro assay using the Z‐LLVY‐AMC substrate to measure chymotrypsin‐like proteasome activity. We observed a reduced proteasome activity in worms that were exposed to the 5MER peptide (Figure 5a,b). While surprising, this result was consistent with our finding that the IIS inhibitor NT219 protects worms from Aβ‐mediated proteotoxicity and reduces proteasome activity (Moll et al., 2016). It is important to note that proteasome activity plays important roles in countering Aβ toxicity and that the 5MER peptide cannot mitigate Aβ proteotoxicity in worms that their proteasome activity was knocked down by *rpn‐6*.*1* RNAi (Figure S4A). Nevertheless, the 5MER peptide does not impair proteasome activity in wild‐type animals as shown by two techniques: an in‐vitro assay (Figure S4B) and the blotting of high‐molecular‐weight ubiquitin conjugates (Figure S4C). This observation implies that this phenomenon of reduced proteasome activity is dependent on the proteotoxic stress of Aβ. In addition, the 5MER peptide did not affect the rate of autophagy in neuroblastoma N2 cells that are not challenged by proteotoxicity (Figure S4D).

Next, we cultured CL2006 worms on *txt‐13* RNAi bacteria and employed the paralysis assay to examine whether TCS is involved in the proteostasis‐promoting mechanism downstream of the 5MER peptide. Our results indicate that the knockdown of *txt‐13* protects from proteotoxicity and that a combination of the 5MER with RNAi towards this gene shows no additive protective effect (Figure 5c). In agreement, a computational analysis has shown that genes that were reported to be components of the TCS mechanism exhibit modulated expression levels upon treatment with the 5MER peptide (Figure S4E). To further test whether the TCS pathway is activated by the peptide, we conducted a paralysis assay using CL2006 worms and RNAi towards either *clec‐41* or *asp‐12*, both needed for TCS activation (O'Brien et al., 2018). We found that the 5MER peptide cannot mitigate proteotoxicity when the TCS is inactivated (Figure S4F).

Since the proteostasis network differentially responds to distinct proteotoxic challenges (Boocholez et al., 2022), we asked whether the 5MER peptide elicits similar gene expression modulations in worms that are challenged by polyQ35‐YFP worms. AM140 were grown on EV bacteria and exposed to 200 μM 5MER peptide at days 1 and 2 of adulthood. An identical group of AM140 worms was served as a control. At day 3 of adulthood, both worm groups were harvested, RNA was extracted, and the relative levels of *nhr‐181*, *lmp‐2*, and *txt‐13*, all of which exhibited modulated expression levels in 5MER peptide‐treated CL2006 worms (Figure 4d,e), were compared by qPCR. Similar expression levels of all three genes were observed in control and treated worms (Figure S5A), suggesting that the 5MER peptide activates different protective mechanisms in the face of distinct proteotoxic challenges.

## 4 DISCUSSION

The failure to develop efficient remedies for Alzheimer's disease (Panza et al., 2019) and the accumulating evidence that AD is not a single disease but a syndrome with various manifestations (Ben‐Gedalya et al., 2015; Szaruga et al., 2015; Vogel et al., 2021) highlight the importance of developing a combinatorial approach for the treatment of this condition. A combined therapy that concurrently modulates different nodes of the proteostasis network bears the potential to harness the mechanisms that prevent the manifestation of AD early in life to postpone disease onset and delay its progression once it has emerged. While short peptides have been previously shown to reduce protein aggregation, including Aβ (Sato et al., 2006), the mechanisms that underlie the counter‐proteotoxic effects of these peptides are largely obscure. In this study, we employed model nematodes to test whether a peptide of 5 amino acids (MTADV), which was shown to mitigate the aggregation of serum amyloid A (Hemed‐Shaked et al., 2021), is capable of suppressing the toxicity of aggregative peptides that underlie the development of neurodegenerative disorders in humans. We discovered that the 5MER peptide alleviates the proteotoxic effects of both the AD‐causing Aβ peptide and of HD‐linked polyQ stretches. The dependency of this protection on TOR signaling (Figure 5di) and proteostasis‐mediating transcription factors (Figure 5dii) predicted that the 5MER peptide modulates gene expression. To scrutinize this hypothesis and identify genes that show modulated expression levels upon treatment with the peptide, we conducted an RNA‐seq experiment. The effect of the 5MER peptide on the worm's transcriptomic landscape (Figure 5diii) was found to be more prominent in worms that express high Aβ levels compared to the outcome seen in their counterparts that express low Aβ levels. Among the genes that showed modified expression, we identified nuclear hormone receptors, known to regulate transcription as well as F‐box proteins. These results raise the question of what counter‐proteotoxic mechanisms are activated by the 5MER peptide and whether it functions as a modifier of transcription in a cell‐autonomous or nonautonomous manner.

Despite the key roles of cellular mechanisms in proteostasis maintenance, the integrity of the proteome is orchestrated at the organismal level by neurons (Prahlad & Morimoto, 2011; Volovik et al. 2014b) and the reproductive system (Moll et al., 2018; Shemesh et al., 2013). This regulation, which involves the activity of neuronal receptors (Maman et al., 2013), is mediated by both neurotransmitters (Tatum et al., 2015) and neuropeptides (Boocholez et al., 2022). The finding that the 5MER peptide protects worms from the toxicity of polyQ‐YFP stretches, regardless of whether they are expressed in neurons or muscle cells, can be explained by two models. One suggests that the peptide migrates between tissues and activates protective mechanisms in cells that are challenged by proteotoxicity, while the other proposes that the 5MER peptide activates mechanisms that function at the organismal level. The reduced expression of *txt‐13*, a gene that encodes a suppressor of TCS that is expressed in muscle cells (Miles et al., 2023), in worms that were treated with the peptide (Figure 4c), and the protection from Aβ proteotoxicity conferred by the knockdown of this gene (Figure 5div), strongly suggest that TCS is activated by the peptide. TCS was shown to promote proteostasis across tissues (O'Brien et al., 2018). Additional support for the theme that the 5MER peptide functions, at least partially, cell‐non‐autonomously is provided by the observation that the intestinal receptors *nhr‐79*, *nhr‐121*, and *nhr‐137* (Cao et al., 2017) show modified expression levels in 5MER‐treated worms (Figure 4c) and by the modulated expression of genes that are involved in signaling in proteotoxicity challenged 5MER‐treated worms (Figure 4b). Together, these observations raise the prospect that the 5MER peptide activates in the intestine a signaling mechanism (Hodge et al., 2022) that communicates with muscle cells and neurons, perhaps via TCS, to enhance proteostasis. Yet, this theme should be further investigated using worms that express TCS‐activating chaperones such as *daf‐21*, in a tissue‐specific manner.

Moreover, the peptide downregulates the expression of *T01D1*.*8*, a gene that is enriched in the ASI/ASJ neurons (Cao et al., 2017), cells that orchestrate proteostasis in the soma (Klabonski et al., 2016). Nevertheless, the possibility that the 5MER peptide promotes proteostasis cell‐autonomously cannot be excluded as it may regulate gene expression within the cell that is challenged by proteotoxicity. It is also possible that the 5MER peptide acts concurrently, cell‐autonomously and non‐autonomously. While our results show that TOR signaling is needed for the peptide to counter proteotoxicity. One key open question is whether the productive system is involved in the 5MER peptide‐mediated protection from proteotoxicity. Further research is needed to fully elucidate which signaling pathways are involved in the mediation of this effect at the cellular and organismal levels.

An additional intriguing question is what activities of the proteostasis network are modulated by the 5MER peptide. We discovered that treatment with the 5MER peptide enhances the aggregation of both Aβ and polyQ35‐YFP (Figures 2 and 5dv). This observation is in line with the requirement for DAF‐16, which was shown to promote protective hyper‐aggregation (Cohen et al., 2006) that sequesters highly toxic oligomers to create large fibrils of lower toxicity (Shankar et al., 2008). However, similarly to other short peptides that were predicted by simulations to reduce aggregation (Jana et al., 2021), the 5MER peptide was found to inhibit the aggregation of serum amyloid A in in‐vitro experiments (Hemed‐Shaked et al., 2021). This apparent contradiction may result from different properties of the 5MER peptide in the absence of ancillary biological molecules in in‐vitro assays that may shape its effect on protein aggregation. An alternative explanation suggests that the peptide's effect on aggregation may be shifted in the face of different Aβ concentrations. In fact, opposing activities are performed in‐vitro by the chaperone HSP104 when the concentration of an aggregation‐prone protein exceeds a certain threshold (Shorter & Lindquist, 2004). In addition, the features of serum amyloid A aggregates may entirely differ from those of Aβ aggregates. Thus, the 5MER peptide may have opposing effects on the aggregation rates of these proteins.

Since protein disaggregation and degradation are also important counter‐proteotoxic activities (Cohen et al., 2006), we asked whether the peptide enhanced the activity of the ubiquitin proteasome system (UPS) and discovered that it reduced chymotrypsin‐like proteasome activity (Figure 5a,b,dvi). This counterintuitive finding is not entirely surprising as we reported previously that proteasomes are not involved in the degradation of Aβ in mammalian cells (Cohen et al., 2006) and that the IGF1 signaling inhibitor NT219 reduces UPS activity in cells (Moll et al., 2016) and mitigates Aβ‐mediated proteotoxicity in worms (El‐Ami et al., 2014). On the contrary, reducing proteasome activity by the knockdown of *rpn‐6*.*1* prevents the 5MER peptide from countering Aβ proteotoxicity (Figure S4A). Therefore, it is possible that the in‐vitro assay shows changes in the total proteasome activity while proteasomes are activated by the peptide in specific tissues of the worm. It may be also possible that not proteasomes but other proteolytic entities, plausibly proteases, digest Aβ in our worms and in other systems. In this regard, the reduced expression of *lmp‐2*, a gene that encodes a membrane protein that is located at late endosomes and lysosomes, upon treatment with the 5MER peptide, predicts that the peptide modulates protein degradation by lysosomes, an organelle that mitigates proteotoxicity and neurodegeneration (Bourdenx et al., 2021). The possible involvement of autophagy and lysosomes in the counter‐proteotoxic effects of the 5MER peptide require further research.

The complexity of neurodegenerative processes and the failure to develop efficient drugs for the treatment of AD infer that a combinatorial approach that concomitantly modulates different nodes of the proteostasis network is likely to preserve proteostasis through late stages of life. The observations that the 5MER peptide protects worms from two highly aggregative peptides, Aβ and polyQ35‐YFP, suggest that it should be considered as a component of such a future counter‐proteotoxic cocktail.

## AUTHOR CONTRIBUTIONS

EC, DN, HES, and RBH designed and initiated this study. NR, LM, HB, RBH, AAS, HES, and EC performed proteotoxicity assays. HES conducted thrashing assays, and NR and LM compared the aggregation rates of Aβ. HES performed the cloning of RNAi plasmids. AZ and IP performed computational analyses of RNA‐seq data. IC and HZ compared proteasome activity levels, constructed plasmids, and conducted western blot assays. EC performed NAGE assays and wrote the manuscript.

## FUNDING INFORMATION

This study was generously supported by the Israel Science Foundation (ISF) (EC#543/21), the Israeli Ministry of Science and Technology (MOST), the United States‐Israel Binational Science Foundation (BSF) (EC#2017241), and the Henri J. and Erna D. Leir Chair for Research in Neurodegenerative Diseases. HES is partially supported by Galmed Pharmaceuticals Ltd., Tel Aviv, Israel.

## CONFLICT OF INTEREST STATEMENT

DN was a consultant of Galmed Pharmaceuticals and an owner of 5MER peptide patent. The other authors declare no competing financial interests.

## Supporting information


Figure S1–S5

Table S1–S4

## Data Availability

The raw and processed NGS files have been deposited at the GEO website and can be accessed at: https://www.ncbi.nlm.nih.gov/geo/query/acc.cgi?acc=GSE230640.
