# Supplementary material for: A short peptide protects from age‐onset proteotoxicity
Source: Aging Cell. 2023 Oct 27;22(12):e14013. doi: 10.1111/acel.14013 (PMC10726816; doi:10.1111/acel.14013)
Supplement: Supplementary file 1 — Figure S1–S5 [file ACEL-22-e14013-s001.pdf]

Supplemental figure 1

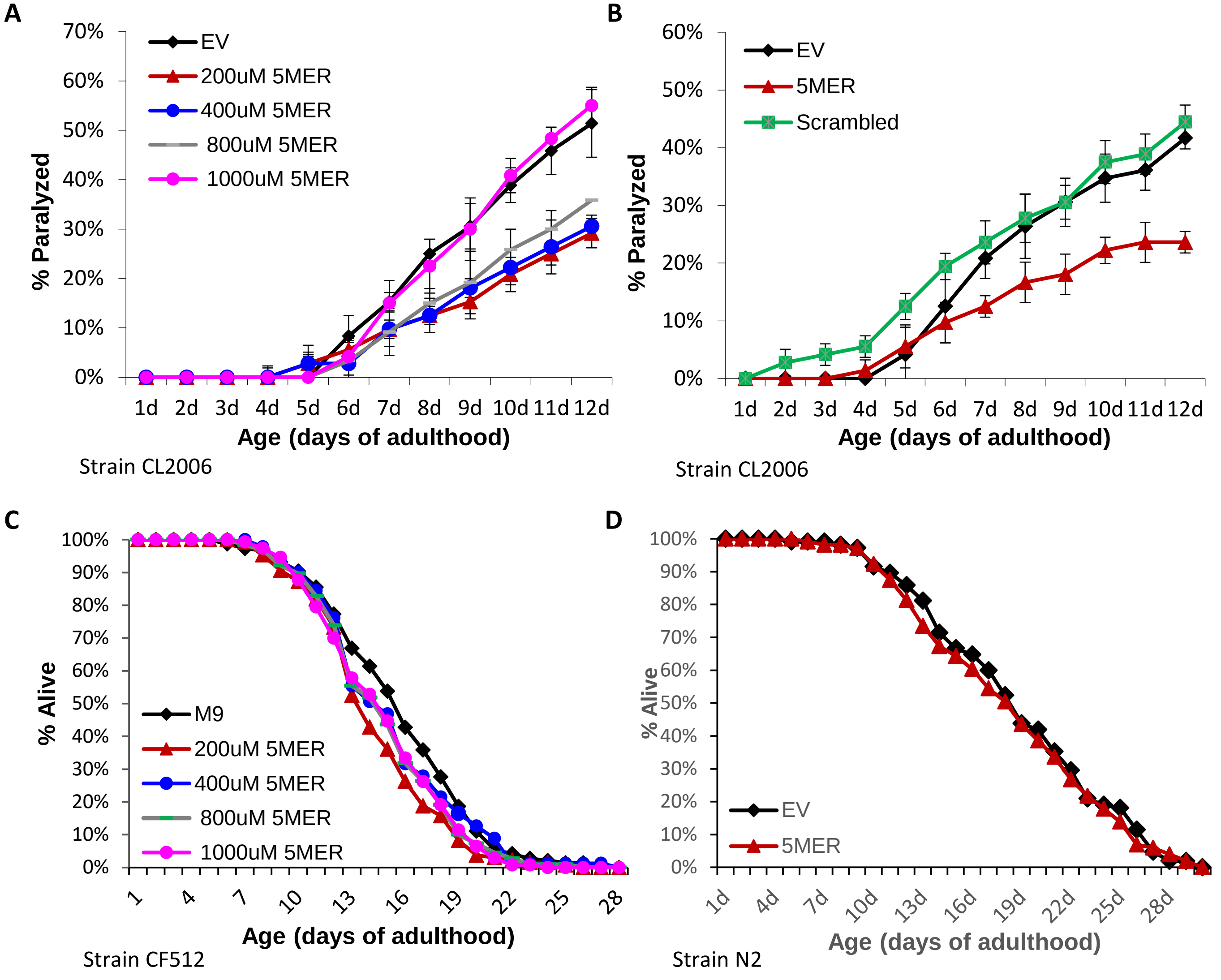

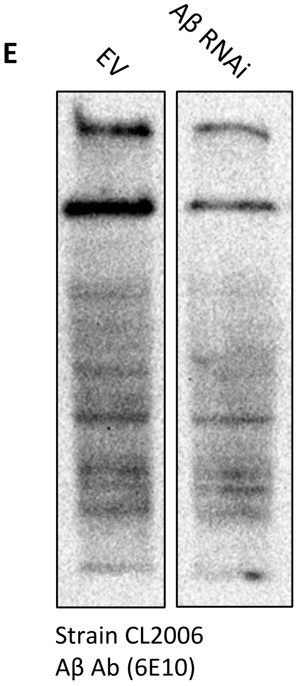

Figure S1: A. A paralysis assay using CL2006 worms indicates that 5MER concentrations higher than 200µM (400, 800 and 1000µM) do not enhance protection from Aβ-mediated paralysis. In fact, the exposure of the worms to 1000µM abolished the protection nediated by the 5MER peptide. **B.** A paralysis assay using CL2006 worms indicated that a scrambled peptide, composed of the same 5 amino acids but in a different order, does not mitigate Aβ-mediated proteotoxicity. **C**. A lifespan assay showed that treatment with the 5MER peptide does not extends lifespan of CF512 worms (C) in various concentrations. **D.** Similarly, treating wild-type worms (strain N2) with 200µM has no effect on lifespan. **E.** A western blot analysis shows a reduction in the level of Aβ in CL2006 worms that were grown from hatching on Aβ RNAi bacteria.

Supplemental figure 2

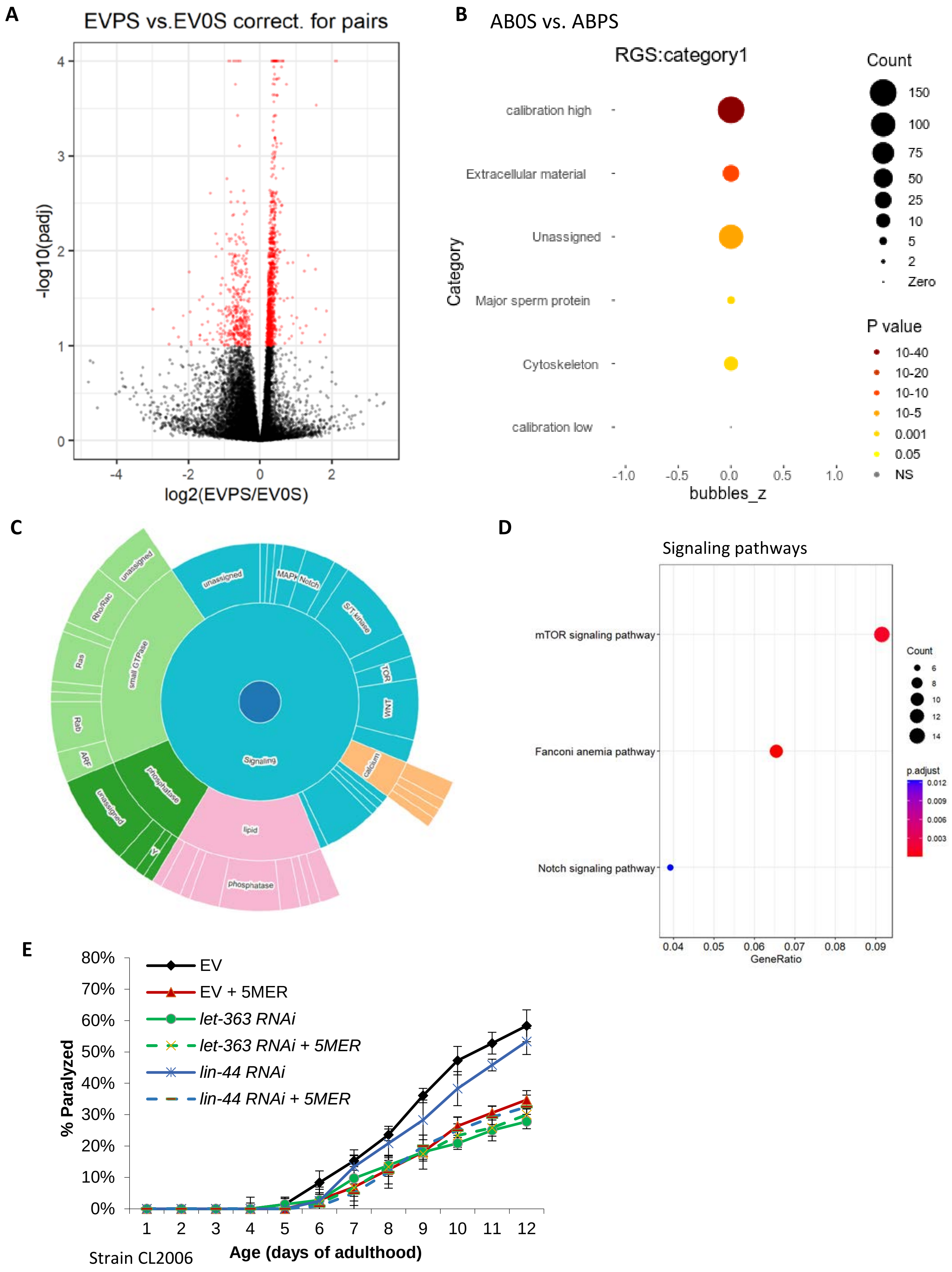

Figure S2: Computational analysis of RNA-seq data. **A.** A volcano plot of 1395 genes that showed significantly differential expression upon treatment with the 5MER peptide in worms that express high Aβ levels (EV0S vs. EVPS). **B.** Wormcat based clustering of genes that exhibit differential expression upon treatment with the 5MER peptide in worms expressing low Aβ levels (AB0S vs. ABPS) show no association with signaling pathways nor with the ubiquitin proteasome system. **C.** Wormcat based analysis of signaling-associated genes that showed modulated expression levels in 5MER peptide-treated worms that express high Aβ levels, indicate that Notch, TOR and Wnt signaling pathways are affected by the 5MER peptide. **D.** Clustering according to involvement in signaling unveiled that TOR signaling is most likely to be affected by the 5MER peptide. **E.** A paralysis assay indicates that the knockdown of *let-363* (encoding the kinase TOR) protects the worms from proteotoxicity and that the 5MER peptide can no further mitigate the toxicity of Aβ. In contrast, the knockdown of *lin-44* results in no reduction of Aβ proteotoxicity. Accordingly, the 5MER peptide protects *lin-44* RNAi-treated worms from this toxicity, indicating that this gene is not required for the 5MER to confer its protective effect.

Supplemental figure 3

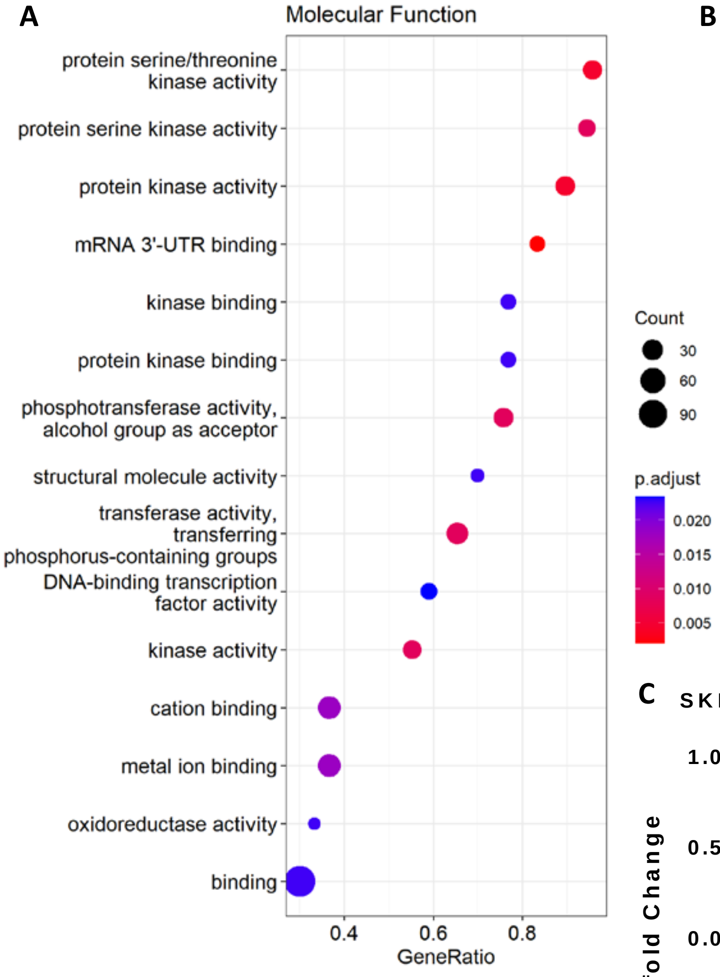

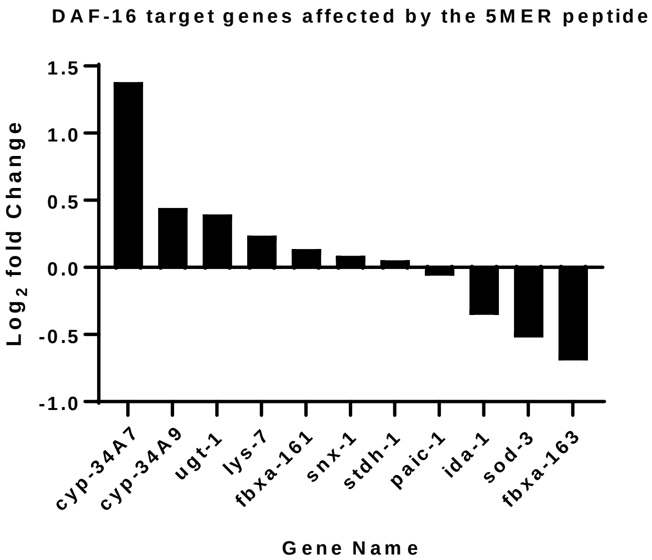

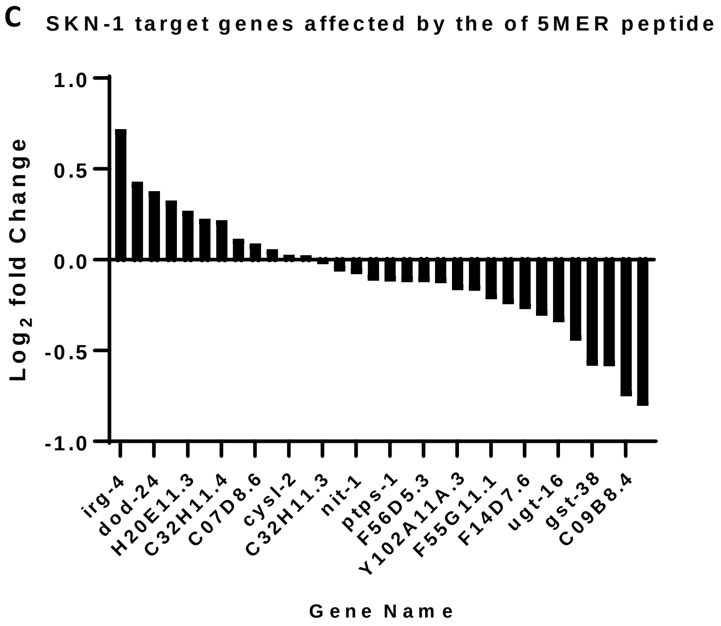

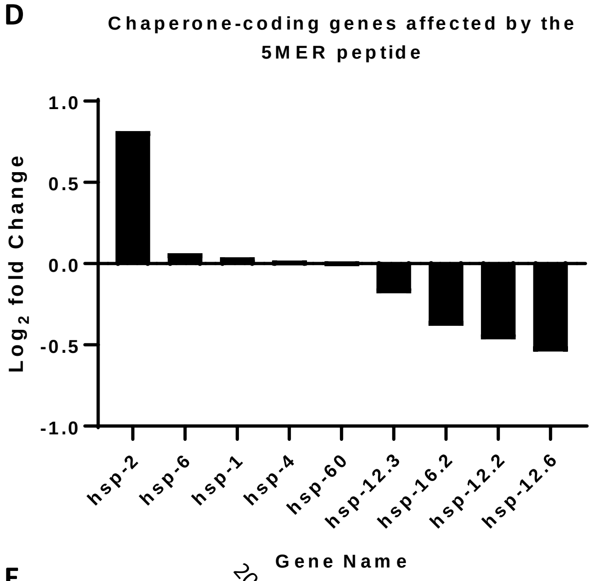

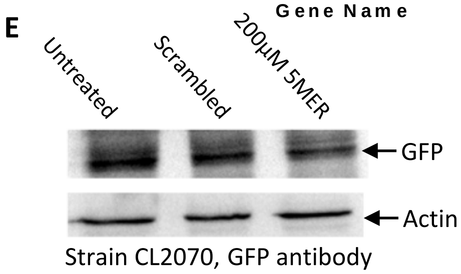

Figure S3: A. Clustering the genes that exhibited significantly ($p<0.05$) modulated expression levels upon treatment with 200µM 5MER peptide according to their molecular functions, indicate that kinase activity and phosphorylation are the most prominent functions. These results support the theme that the 5MER peptide induces signaling. **B-D.** The effect of the 5MER peptide on the expression levels of known target genes of DAF-16 (B), SKN-1 (C) or on the expression of genes coding for a subset of chaperones (D). **E.** Treatment with the 5MER peptide lowers the level of GFP driven by the *hsp-16.2* promoter.

Supplemental figure 4

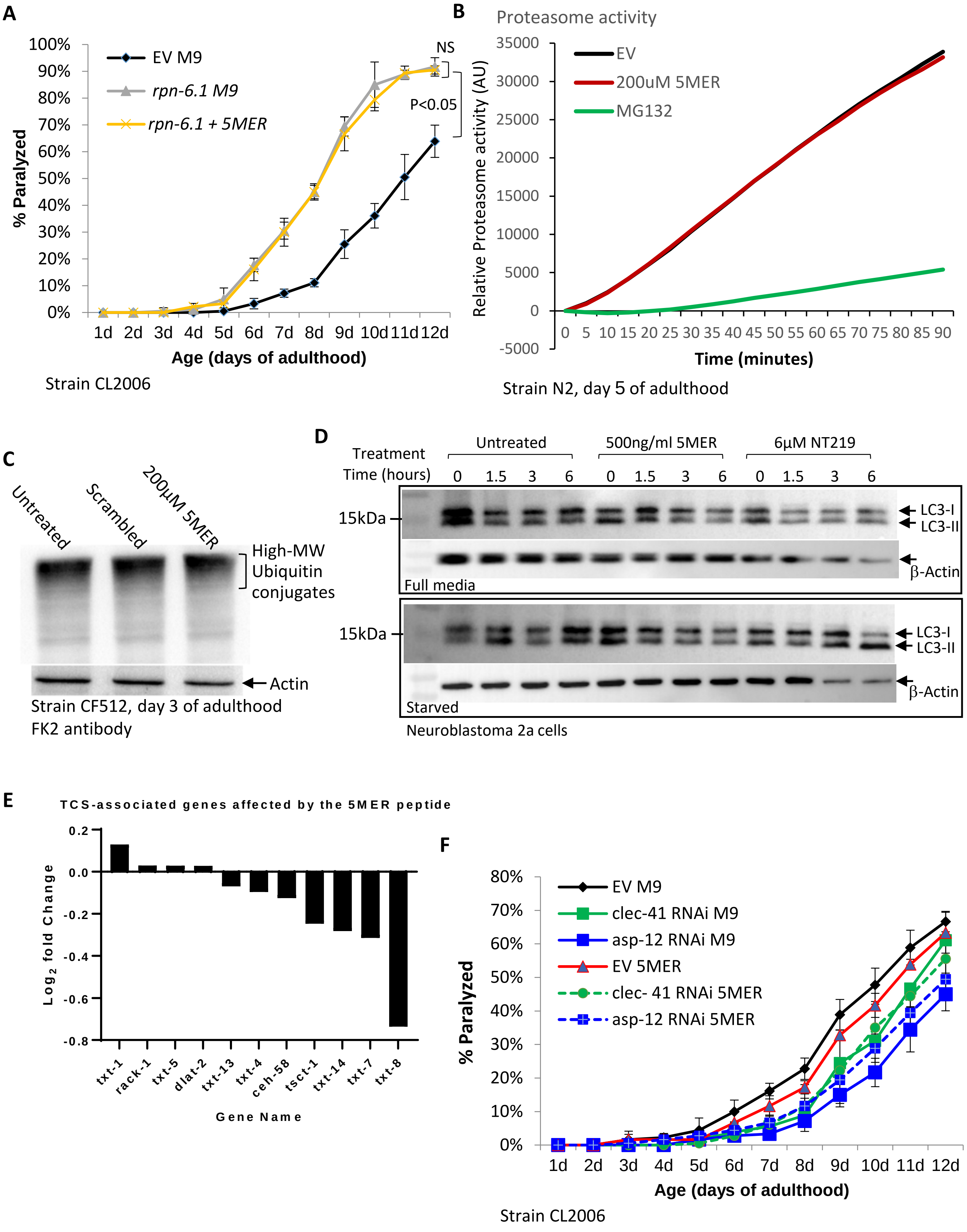

Figure S4: A. A paralysis assay indicates that the knockdown of *rpn-6.1* (needed for UPS activity) aggravates Aβ-mediated toxicity. The 5MER peptide cannot rescue this deleterious phenotype. **B.** *In-vitro* proteasome activity assay indicates that the 5MER peptide does not affect chymotrypsin-like activity of 5 days old wild-type worms. **C.** Similarly, the 5MER peptide does not inhibit proteasome activity in 3 days old CF512 worms as judged by the blotting of high molecular weight ubiquitin conjugates. **D.** The 5MER peptide does not enhance autophagy in neuroblastoma 2a cells. **E.** The expression levels of certain TCS-associated genes are modulated by the 5MER peptide as seen in our NGS data. **F.** Rate of paralysis of CL2006 worms that were treated with RNAi toward either *asp-12* or *clec-41* indicate that the 5MER peptide cannot further protect these animals from proteotoxicity.

Supplemental figure 5

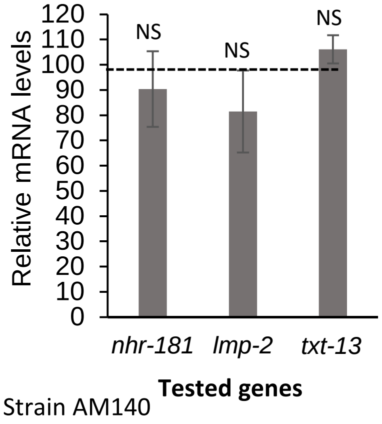

Figure S5: . A. qPCR using AM140 worms and primer sets toward *nhr-181, lmp-2 or txt-13* indicates that the 5MER does modify the expression levels of these genes in worms that are challenged by the proteotoxicity of polyQ35-YFP.
