# Supplementary material for: A short peptide protects from age‐onset proteotoxicity
Source: Aging Cell. 2023 Oct 27;22(12):e14013. doi: 10.1111/acel.14013 (PMC10726816; doi:10.1111/acel.14013)
Supplement: Supplementary file 2 — Table S1–S4 [file ACEL-22-e14013-s002.pdf]

Supplementary table 1: worm strains used in this study

Strain	Genotype	Source
N2	Wild type, Bristol	CGC
CF512	fer-15(b26)II;fem-1(hc17)IV	CGC
CL2006	pCL12 (unc-54/human Aβ peptide 1-42 minigene) + pRF4	CGC
AM140	rmIs132 [unc-54p::Q35::YFP]	CGC
AM716	rmIs284 [pF25B3.3::Q67::YFP]	A gift of Prof. Richard Morimoto
CF1934	daf-16(mu86); muIs109[Pdaf-16::gfp::daf-16cDNA + Podr-1::rfp]	A gift of Prof. Andrew Dillin
CL2070	dvIs70 [hsp-16.2p::GFP + rol-6(su1006)]	CGC

Supplementary table 2: primers used for cloning of RNAi plasmids

Gene	Forward	Reverse
nhr-58	Includes XhoI site<br>CAATCTCGAG**ATTCTTCTCCGTCACTCG**	Includes NheI site<br>ATTGCTAGC**TTCTGAATAATCGGACATAGGG**
txt -13	Includes XhoI site<br>CAATCTCGAG**AGAGATCATTCGAGGAAACC**	Includes NheI site<br>ATTGCTAGC **ACGCGTATACATACAAACG**
let-363	Includes XhoI site<br>TTCGGTACC**TGCATGTATGTCAATGGTTGG**	Includes KpnI site<br>ATGCTCGAG**TCAATGGAGATGTTGCTTGC**
lin-44	Includes XhoI site<br>TTCGGTACC**CAACCCTTGAGCACATTACC**	Includes KpnI site<br>ATGCTCGAG**AATCCTTACAGACCAGAT TGC**
clec-41	Includes AgeI site<br>ATGACCGGT**AGTTGGAACACAGTGTGG**	Includes NheI site<br>ATGGCTAGC **GGCTGAGTAACATCAGTGG**
asp-12	Includes AgeI site<br>ATGACCGGT**TTCCAGGAAGTAACTGCG**	Includes NheI site<br>ATGGCTAGC**AGACGAAATGTGAGAGGC**

Supplementary table 3: primers used for qPCR

Gene	Forward	Reverse
txt-13	GCTGCTGCGAGTCCTACATT	CCTCAGTATCTTGGCGACGG
nhr-58	TTGTCGATACGCGGAGAATG	GTGGTGTAGAGCGCCAAA
nhr-181	ATTTCCTGTCGAGCCCACAT	TCGTGTTGAAGGCGGAC
lmp-2	TGGCTGGACAATTGACTTTGC	CGGGAAATGCAGTTGGATCG
cyn-17	ACAACGACTACAATGGCA	CTGGTGTTGGCTTTTGACGG

Supplementary table 4: Lifespan data

Strain	Treatment	# included	Mean LS ± StEr	T-test
CF512	M9	143/150	19.02±0.434	
	200uM 5MER	148/150	18.64±0.426	p=0.268
CF512	M9	145/160	16±3.93	
	200uM 5MER	133/160	14±3.99	p<0.001
	400uM 5MER	130/160	15±3.63	p>0.05
	800uM 5MER	110/160	15±3.80	p=0.014
	1000uM 5MER	140/160	15±3.66	p<0.006
AM140	M9	112/120	13.2±0.333	
	200uM 5MER	106/120	13.96±0.278	p<0.05
	400uM 5MER	108/120	13.2±0.313	p=0.29
N2	M9	105/120	18.91±0.545	
	200uM 5MER	101/120	18.93±0.593	P=0.19
